# Beclin-1- mediated autophagy may be involved in the elderly cognitive and affective disorders in streptozotocin-induced diabetic mice

**DOI:** 10.1186/s40035-016-0070-4

**Published:** 2016-12-12

**Authors:** Zhu-Fei Guan, Xiu-Ling Zhou, Xiao-Ming Zhang, Yu Zhang, Yan-Mei Wang, Qi-Lin Guo, Gang Ji, Guo-Feng Wu, Na-Na Wang, Hao Yang, Zhong-Yu Yu, Hou-Guang Zhou, Jing-Chun Guo, Ying-Chao Liu

**Affiliations:** 1 Department of Geriatric Neurology, Huashan Hospital, Fudan University; National Clinical Medicine Research Center for Age-related Diseases, 12 Middle WuLuMuQi Rd, Shanghai, 200040 China; 2 State Key Laboratory of Medical Neurobiology, Department of Neurobiology, School of Basic Medical Neurobiology, Department of Neurobiology School of Basic Medical Science, Shanghai Medical College, Fudan University, 131 DongAn Rd, Shanghai, 200032 China; 3 Department of Ultrasonics, Huashan Hospital, Fudan University, Shanghai, 200040 China; 4 Department of EmergencyNeurology, Guiyang Medical University, Guiyang, 550004 China; 5 Department of Neurosurgery, Shandong Provincial Hospital, 5 Latitude and 7 longitude Rd, Jinan, 250021 China

## Abstract

**Background:**

Diabetes is the most common metabolic disease with many chronic complications, and cognitive disorders are one of the common complications in patients with diabetes. Previous studies have showed that autophagy played important roles in the progression of metabolic syndrome, diabetes and other diseases. So we investigated whether aged diabetic mice are prone to be associated with the cognitive and affective disorders and whether Beclin-1-mediated autophagy might be involved in thepahological process.

**Methods:**

High-fat diet/streptozotocin (STZ) injection-induced diabetic C57 mice were adopted in this study. Cognitive disorders were detected by Morris water maze and fear conditional test. Affective disorders were detected by tail suspension test and forced swimming test. Magnetic resonance imaging was applied to observe changes of morphology and metabolism in the brain. The 18 F-fluorodeoxyglucose positron emission tomography (FDG-PET) was used to assess metabolism changes in the brain of aged diabetic mice. Autophagy were evaluated by Beclin- 1, LC3II/I and P62, which were detected by western blot analysis and observed by electron microscopy.

**Results:**

1. Compared with control group, diabetes mice showed significantly decreasing abilities in spatial memory and conditioned fear memory (all *P* < 0.05), and increasing tendency of depression (*P* < 0.05). 2. MRI showed that the majority of elderly diabetic mice were associated with multiple cerebral small vessel disease. Some even showed hippocampal atrophy, ventricular dilatation and leukoaraiosis. 3. FDG-PET-CT discovered that the glucose metabolism in the amygdala and hippocampus was significantly decreased compared with normal aged mice (*P* < 0.05). 4. Electron microscopy found that, although autophagy bodies was not widespread, and there was no significant difference between the two groups, yet compared with normal aged mice, apparent cell edema, myelinated tow reduction and intracellular lipofuscin augmentation existed in elderly diabetic mice brain. 5. The level of p62 was increased in the STZ-induced diabetic mice hippocampus and striatum, and beclin1 protein expression were significantly decreased in diabetic mice hippocampus compared with normal aged mice (*P* < 0.05). There was a upward trend of the ratio of LC3II/I in hippocampus, cortex and striatum, but no statistically difference between the two groups.

**Conclusion:**

Compared with normal aged mice, diabetic aged mice were apt to cerebral small vessel disease and associated with cognitive and affective disorders, which may be related to the significantly reduced glucose metabolism in hippocampus and amygdala. Beclin1 mediated autophagy in hippocampus probably played an important role in cognitive and affective disorders of STZ-induced aged diabetic mice.

## Background

Diabetes is the most common metabolic disease with many chronic complications. Studies on animal models and clinical diabetic patients have revealed that abnormalities appear in both chemical and ultra-structural levels in brain and in neurological behaviors including learning/memory disability and the increasing risk in depression [1]. Oxidative stress [2–7], chronic inflammation [8–10] and autophagic imbalance [11–14] are considered to be most likely causes to induce brain dysfunction [15–17]; however, whether autophagic imbalance is involved in diabetes-related cognitive and affective disorders remains unclear.

Autophagy, a self-eating mechanism, is a crucial cleaning system for aggregated proteins and dysfunctional organelles [18]. It is linked to the degradation of damaged materials by acid hydrolases within the lysosomal system. There is mounting evidence that the control of autophagocytosis is impaired in many neurodegenerative diseases, which display abnormal protein aggregation [19–21]. There are three different pathways of autophagic uptake and processing of cellular constituents, i.e., macro-, micro- and chaperone-mediated autophagy [22]. Beclin1, an adaptor protein via its interacting proteins called the Beclin1 interactome, can either stimulate or suppress the onset of autophagy. Beclin1 is one of the core elements to regulate autophagy. The expression level and post-translational modifications of Beclin1 interactome components can crucially control the degree of cellular autophagy.

Up to date, few researches have investigated the relationship between diabetes-related cognitive and affective disorders and Beclin1-mediated autophagy. The main purpose of this study was to verify whether long-term diabetes could induce cognitive and affective disorders in elderly mice, and to explore whether changes of Beclin1 protein and autophagy could affect this pathogenesis.

## Methods

### Animal grouping

Male C57BL/6 mice were purchased from ProMedican in Shanghai, China. Mice were raised under a 12-h light/dark cycle and humidity- and temperature-controlled environment with adlibitum access to water and standard laboratory chow. All the animal experiments were approved by Fudan University Animal Care and Use Committee. The 3-weeks aged mice were randomly divided into a control group (*n* = 12) and a diabetic group (*n* = 12).

### Model making

**Fig. 1:**
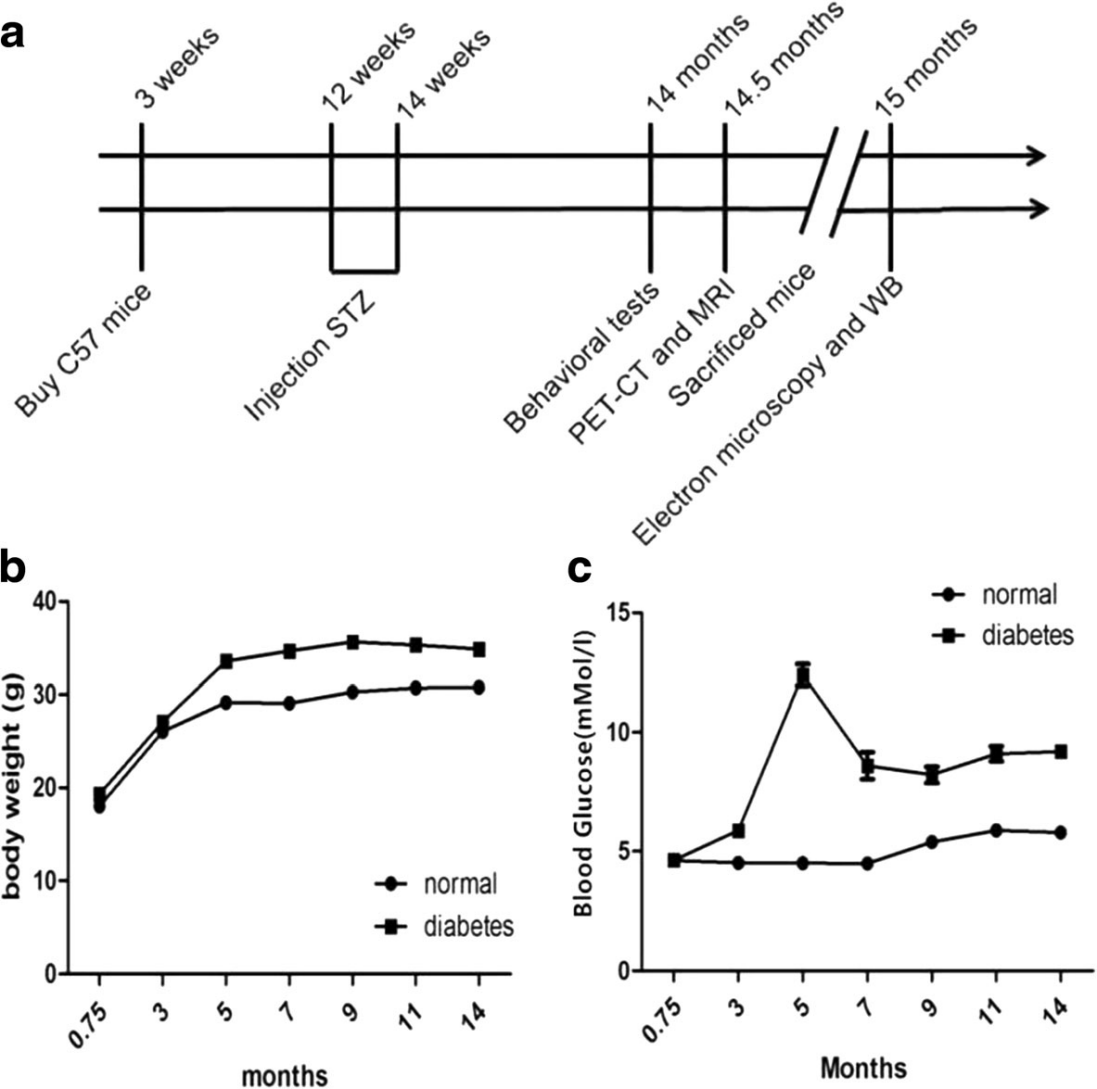
HFD/STZ treatment upregulated body weight and blood sugar in mice. **a** Experimental time-flow diagram. The body weight (**b**) and blood glucose (**c**) of mice were measured before death. Blood samples were collected from tails of mice under fasting condition (*P < 0.05, *n* = 12 for each group)

Control mice were given with normal diet. The model mice were given high fat diet (HFD, Shanghai SLAC company) for 12 weeks. After that, 45 mg/kg Streptozocin (STZ, Enzo life sciences) were intraperitoneally injected for two weeks. Blood glucose in caudal venous of mice were detected after STZ injection. Mice with random blood glucose >11.1 mmol/L were considered as diabetic mice. Then the control and diabetic mice were respectively raised with normal diet and HFD (Fig. 1a).

### Morris water maze test

Learning and reference/working memory were evaluated by the Morris water maze test as previously described [23]. Groups were blinded to the examiners. In brief, swimming paths were video-tracked with a camera fixed on the ceiling of the room and analyzed by the software (Coulbourn, USA). A training session was carried out before the hidden platform test sessions. Mice were given 60 seconds free swimming and guided to climb onto the hidden platform and allowed to remain there for 30 seconds before returning to their cages. On the hidden platform test, the mice had 5 sessions at 20-min intervals per day on the following 4 consecutive days (day 1 to 4). During each session mice were released from randomly assigned 3 starting points and swam for 60 s. On the probe test at day 5, the hidden platform was removed and the mice swam freely for 60 s. The number of times the mice crossed the original platform location was recorded. On the visible platform test which was performed after the probe test on day 5, the platform was elevated 5 cm above the water surface level and placed in a different position. The mice were given four sessions of a visible trial with an inter-session.

### Conditioned fear test

Conditioned fear (fear conditioning) formation is not only a process of learning and memory, but also the formation of an emotional process. The Study on the fear of the fear model used in the rodent fear memory, and the freeze is considered to be an objective indicator of fear [24].

Mice (14-months old) were subjected to fear conditioning test. Fear conditioning test was performed as described previously [25]. Briefly, a mouse was placed in a plexiglas training chamber whose floor had a stainless grid for shock delivery. After a 3-min baseline exploratory period in the chamber, mice received 3 tone (2000 Hz, 90 db)–shock (0.7 mA, 2 s) pairings separated by 1 min. Each training chamber was cleaned with 95% ethyl alcohol before the placement of a mouse and was illuminated only with a 10 W bulb in a dark experimental room. Twenty-four hours after the training session, mouse was placed again in the training chamber for 8 min without tone and foot shock. Each animal’s freezing behavior was scored every 8 s during the 8 min observation period. The percentage of time in freezing behavior was calculated using the formula of 100*f/n, where f was the number of freezing events in the 8 min and n was the total number of 8-s observation period in 8 min. One hour later, the mouse was placed in a new chamber without a stainless floor grid. This chamber was cleaned with the lemon fresh pine-sol each time after use and room light was turned on during the test. After a 3-min exploratory period in this new chamber, a 30-s tone (2000 Hertz, 90 db) was applied. The mouse was then left in the chamber for another 1 min. The percentage of time in freezing was calculated by the formula of 100*f/30, where f is the total freezing time during the 30-s observation period.

### Tail suspension test

Tail suspension test was performed as described previously [26]. Briefly, animals were suspended by the tail from a ledge with adhesive tape (approximately 1 cm from the tip of the tail). The distance between the tip of the tail of the mouse and the floor was approximately 30 cm. Each animal was partitioned to avoid interference during the test. Immobility was defined as the absence of movement for 6 min. Each mouse in the test was recorded by a videocamera and scored by a blinded experimenter.

### Forced swimming test

Forced swimming test was performed as described previously [27]. Briefly, animals were placed in an open cylindrical container (total volume, 2500 mL; height, 20 cm; diameter,14 cm) filled with 10 cm of water (25 °C). Immobility was defined as mouse ceasing struggling, remaining floating motionless in water, and making only movement necessary to keep its head above water. The duration of immobility in the last 4 min of the (total) 6 min of swimming time was recorded with a videocamera and scored by a blinded experimenter.

### Magnetic resonance imaging scanning

We use Bruker 3.1 7 T superconducting magnetic resonance imaging with the diameter of the 47 mm microscope coil to do mouse MR imaging study. Before MR scan, the mice were anesthetized with isoflurane. After that, the mice were fixed on the fixed frame, and the T1, T2 and enhanced scan were performed. Scanning parameters: echo time 20s; repetition time 2000 ms; band width 333333.3hz; layer 16; layer thickness 0.8 mm; image size 64; scanning field of view 21600 ~ 15000 mm.

### PET/CT scanning

Prior to the PET scans, the mice were fasted for 12 h. Each mouse was injected approximately 0.5 mCi (18.5 MBq) 18 F-FDG in less than 0.5 ml via tail vein. The injection was completed in less than 1 min and the injection site was pressed for 30 s to prevent leakage. The exact injection time and the radio activity of the syringe both before and after the injection, together with their measure time, were recorded for dosage calibration. After an uptake period of 40 min, the mouse was anaesthetized with agasmixture of 1% isoflurane and oxygen (1 L/min) and then prone positioned in the gantry with a mask covering its mouth to ensure continuous gas inhalation throughout the scan. The PET signals were acquired from approximately 45 min post-injection for 10 min inaInveon MM micro PET/CT scanner (Siemens Co., Ltd, Knoxville, TN, USA) designed for high resolution imaging of small laboratory animals. The following CT scan was performed for localization and attenuation correction. The raw PET data were binned into a single frame, and the multiple planar reconstruction (MPR) images were obtained by OSEM3D/MAP reconstruction methods with iterations of OSEM3D = 2 and iterations of MAP = 18. The final voxel size was 0.776 × 0.776 × 0.796 mm^3^, and the matrix was 128 × 128 × 159. PMOD 3.4 software (PMOD Technology, Switzerland) was used to analyze the images. Automatic rigid matching was performed after rough manual co-registration in the PFUS module. A Gaussian kernel with full width of half maximum (FWHM) of 0.6 mm × 0.6 mm × 0.6 mm was used to smooth the image during normalization. After that, Mirrione atlas was overlapped on the normalized PET images so as to obtain the SUV mean of all 19 mouse brain regions in this standardized template. Finally, each regional SUV mean was divided by that of the brain stem to derive an SUVR for statistical analysis purpose.

### Transmission electron microscopy

Transmission electron microscopy (TEM, PHILIPS CM-120, Netherlands) Brain tissues were perfused with 2.5% glutaraldehyde perfusate (25% glutaraldehyde and 0.2 M phosphate buffer with 3mMMgCl2, pH 7.4), followed by fixation with 2.5% glutaraldehyde as previously described [28]. And then concentional of fixation solution, dehydrated, embedded in paraffin, sliced and 3% uranyl acetate and lead citrate double staining. The CM-120 PHILIPS was observed and photographed under transmission electron microscope.

### Western Blot

Western Blot was performed as described previously [29]. The hippocampus tissues from mice were homogenized using RIPA (20mgorganizationwith 200 μl RIPA). The tissue lysates were centrifuged at 12,000 rpm for 5 min and the supernatant were collected to determine the protein concentrations by a bicinchoninic acid protein assay (Beyotime Institute of Biotechnology, China). Membranes were reprobed with an antibody specific against GAPDH as an internal control. The specific primary antibodies included: a rabbit polyclonal antibody for BECN1 (1:500; SantaCruz, USA), LC3 (1:200; cell signaling technology, USA), p62 (1:3000, abcam, England), and GAPDH (1: 2000, Boster, China). At last, X-ray films were used to Photosensitive, developing and fixing in dark room.

### Statistical analysis

All statistical analysis was performed using PRISM software (GraphPad, La Jolla, CA). All data were represented as mean ± SEM and analyzed by student *t*-test using treatment (diabetes vs.control). *P* values less than 0.05 were considered significant.

## Results

### HFD/STZ treatment induces upregulation in both body weight and blood glucose level in mice

**Fig. 2:**
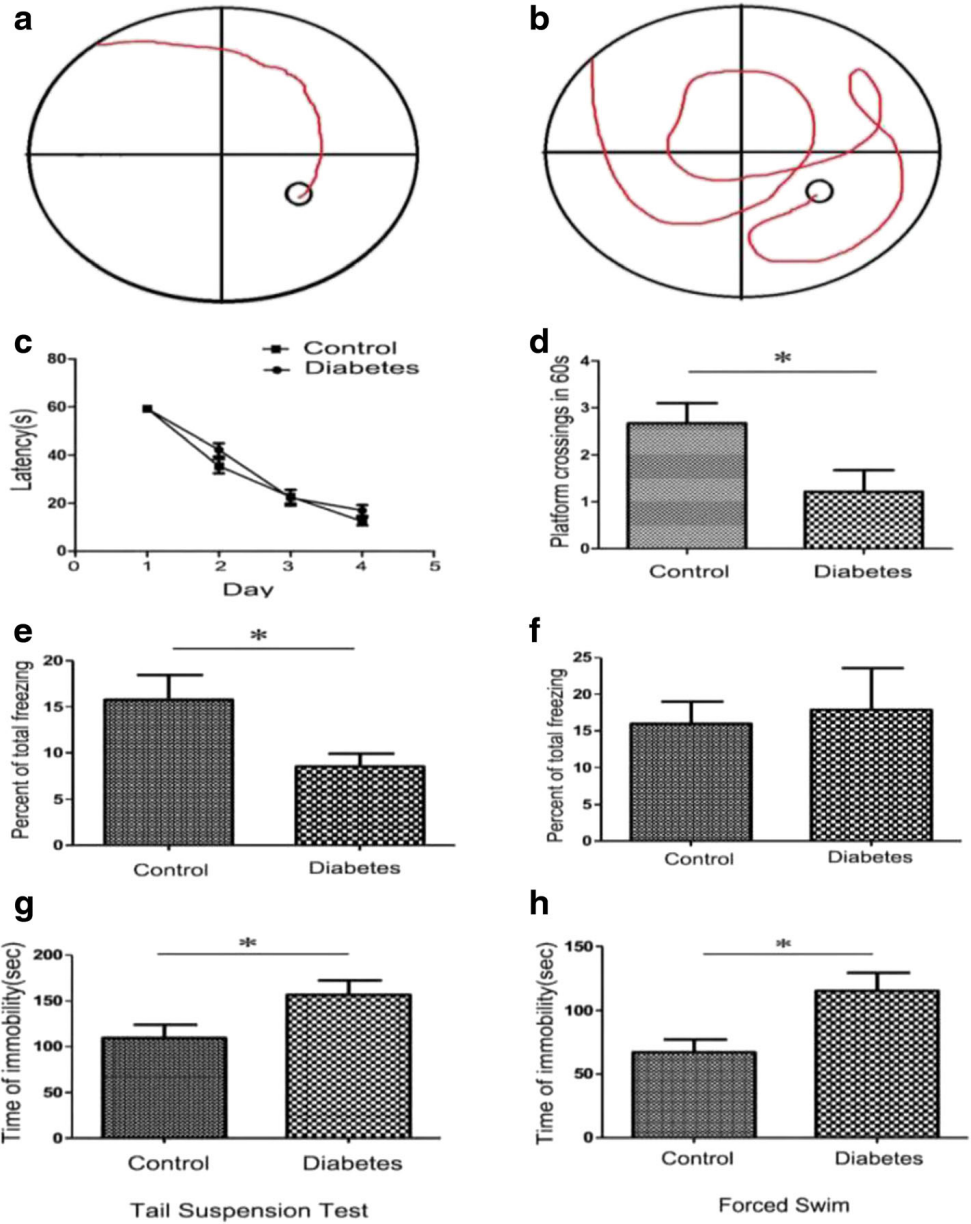
HFD/STZ treatment casused cognitive impairment and depressive symptoms in mice. **a** and **b**, typical swimming traces of control mice (**a**) and diabetic mice (**b**) in Morris watermaze test. **c**, No difference were found between the two groups in the latency time during training trials. **d**, During testing phase, the frequency of platform-crossing in diabetic mice decreased remarkably when compared with control mice. (*P < 0.05, *n* = 12 for each group). Gand F, cued and contextual fear memory was evaluated using the fear conditioning test. Percent of time in total freezing during the contextual test (**e**) and tone test (**f**) was represented. (*P < 0.05, *n* = 12 for each group). Gand H, effects of diabetes on immobility time in the tail suspension test (**g**) and the forced swimming test (**h**). The immobility time in diabetes group was significantly higher than that of control group. (*P < 0.05, *n* = 12 for each group)

Before grouping, all the mice had similar body weight and random blood glucose levels. After STZ injection, the level of blood glucose in HFD/STZ-treated mice was significantly increased till 14 months later. As showed in Fig. 2, HFD/STZ-treated mice showed significant rise in both body weight (Fig. 1b, *P* < 0.05) and blood sugar level (Fig. 1c, *P* < 0.05) compared with those in normal diet-treated control mice. These results indicated that HFD/STZ-treatment successively induced diabetic mice model in 14-month-aged elderly mice.

### Cognitive impairment and depressive symptoms in elderly diabetic mice

We performed Morris water maze test to evaluate the spatial acquisition ability. As showed in Fig. 2, the number of platform crossing significantly decreased in diabetic mice (Fig. 2d, *P* < 0.05), indicating that HFD/STZ-induced diabetes induced remarkable reduction in the spatial learning/memory ability.

In the fear conditioning test, we assessed changes in cued and contextual fear memory in diabetic mice. As showed in Fig. 2e, when compared with control mice, the percentage of time of total freezing behavior decreased significantly in diabetic mice (*P* < 0.05). For the tone test, no obvious difference was observed between two groups, indicating that HFD/STZ-induced diabetic mice had reduction in fear conditioning memory ability.

By using tail suspension and forced swimming, we also detected the depressive status in the mice. As showed in Fig. 2g and h, the time of ‘immorbility’ in diabetic mice remarkably increased during both tail suspension test (Fig. 2g) and forced swimming test (Fig. 2h).

These results indicated that abilities of HFD/STZ-induced diabetic mice decreased in both spatial memory acquisition and fear conditioning memory. Meanwhile, these diabetic mice represented more severe depressive behaviors than the control mice.

### Glucose metabolism reduction and structural abnormalities in the amygdala and hippocampus of elderly diabetic mice

**Fig. 3:**
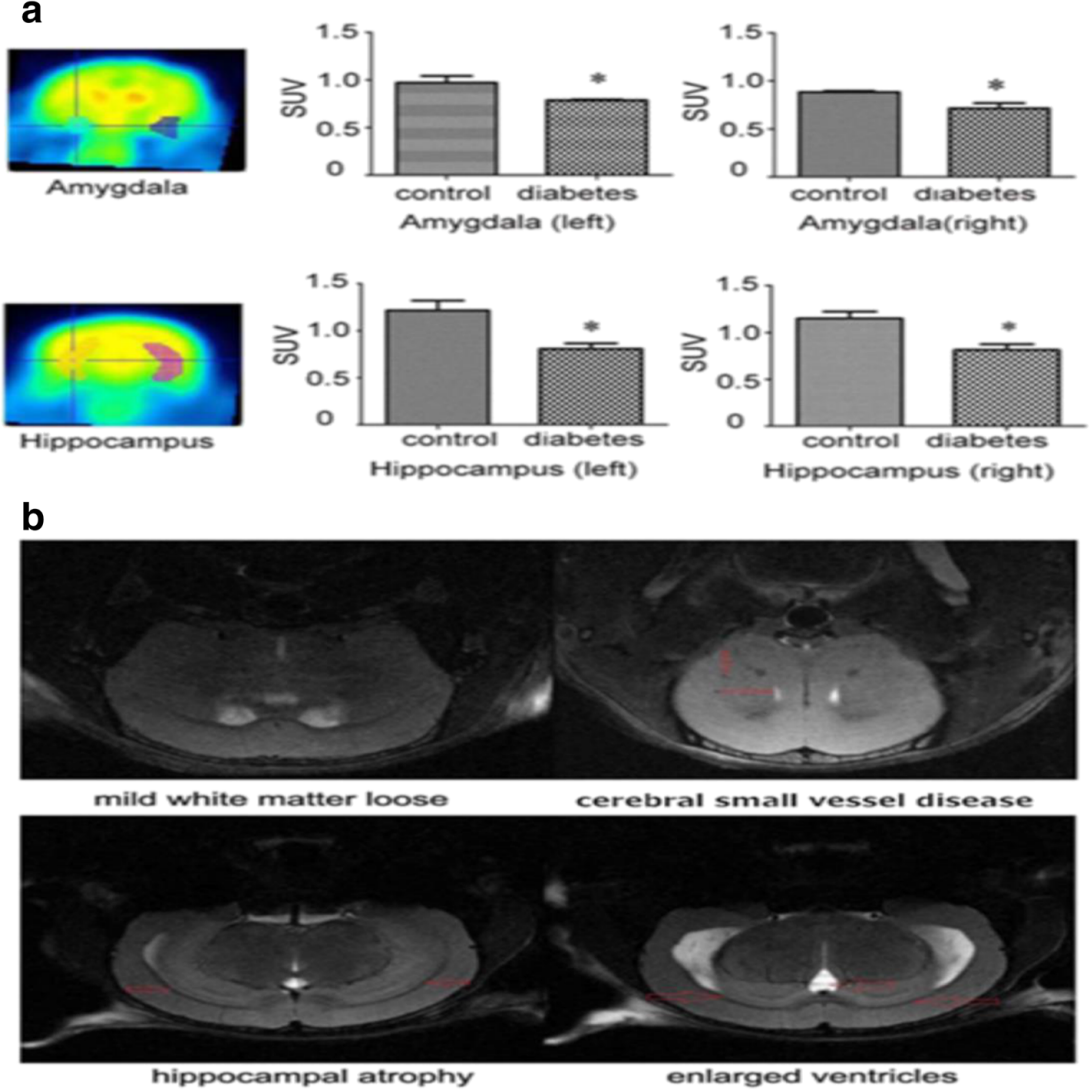
**a** Standard uptake value (SUV) of FDG in the Hippocampus and Amygdala in diabetes group and control group. The SUV of FDG in diabetes group were much less than those in the control (*P < 0.05, *n* = 3 for each group). **b** Magnetic resonance imaging showed mild white matter loose, cerebral small vessel disease, hippocampal atrophy and ventricle enlargement in the brain of diabetic mice

To investigate whether regional functional changes exist in the brain of diabetic mice, we further evaluated the FDG uptake ability by using PET-CT scaning. As showed in Fig. 3a, the standard uptake value of FDG in both hippocampus and amygdala were much less in diabetic mice than those in control mice (*P* < 0.05).

Magnetic resonance imaging showed that the majority of elderly diabetic mice were associated with multiple lacunar infarction which indicated cerebral small vessel disease. Some even appeared hippocampus atrophy, ventricle enlargement in parts of elderly diabetic mice (Fig. 3b).

**Fig. 4:**
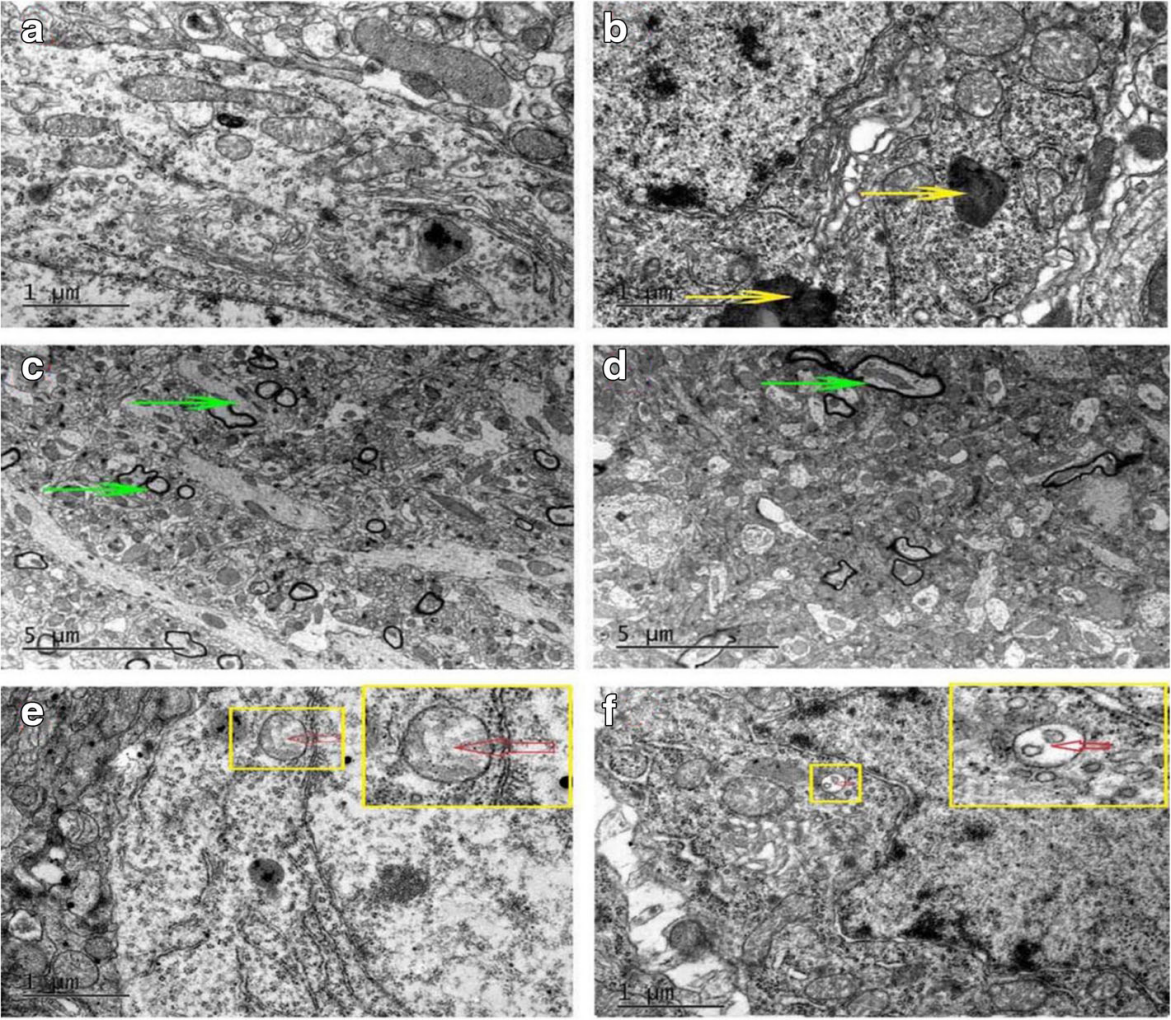
Alterations in hippocampus ultrastructure was examined by transmission electron microscope. Autophagosomes (*red arrow*) existed in both control and diabetic mice were no significantly decrease in diabetes mice. Diabetes mice have more lipofuscin (*yellow arrow*) and fewer myelinated nerve fibers (*green arrow*) in the hippocampus. **a**, **c**, **e** were control groups and (**b**, **d**, **f**) were diabetes groups

We also performed electron microscopy to observe the ultrastructural changes in the hippocampus. As showed in Fig. 4, mice in diabetic group appeared a slight increase without significant differences in autophagosome, but there were more intracellular lipofuscin and less myelinated nerve fibers in diabetic hippocampus, in accompanied with apparent hippocampal edema.

These results suggested that the brain function reduction and structural abnormalities existed in the amygdala and hippocampus of elderly diabetic mice.

### Autophagy was upregulated in the hippocampus, cortex and striatum of elderly diabetic mice

**Fig. 5:**
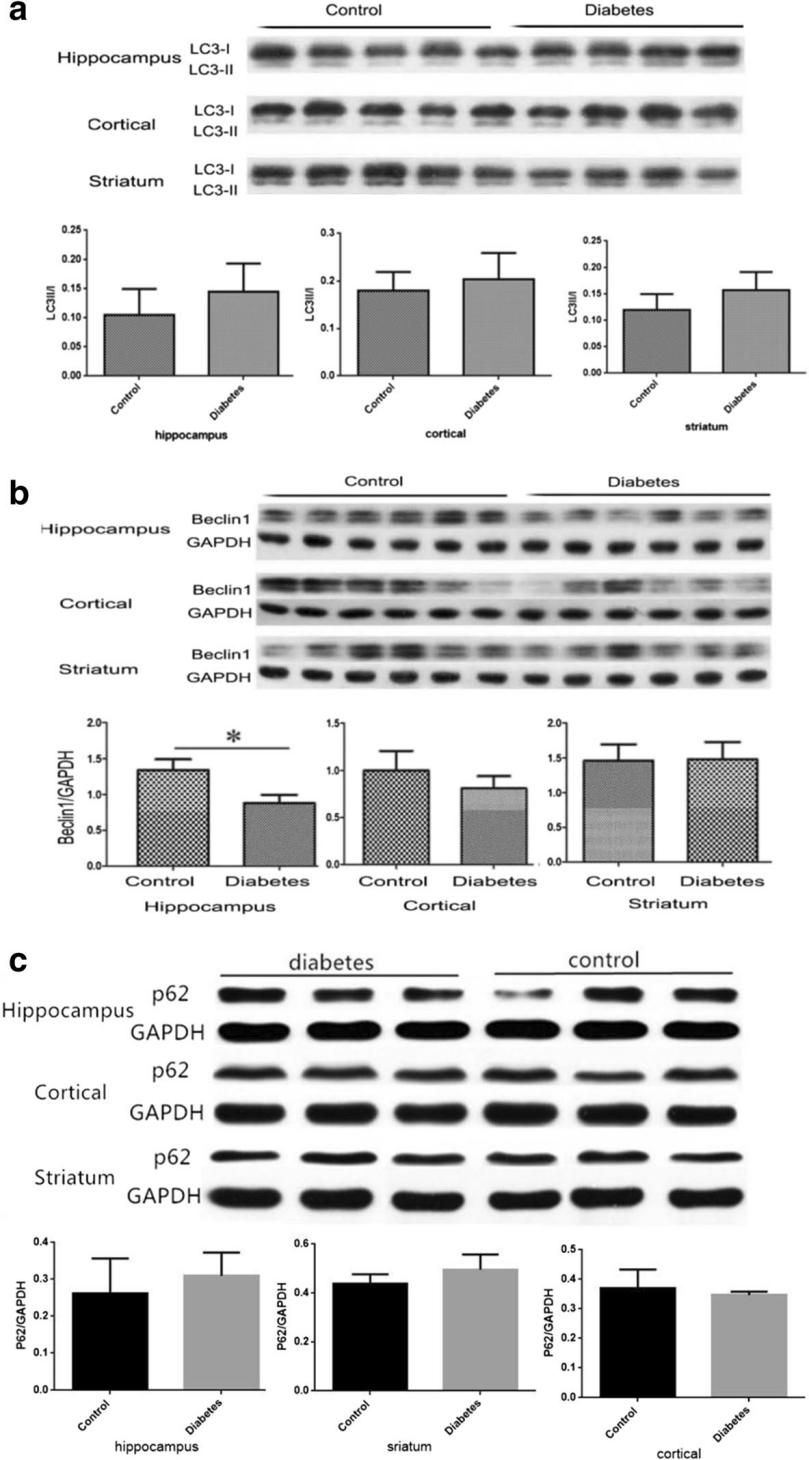
**a** The protein levels of LC3II/LC3I in Hippocampus, Cortex and Striatum were measured by Western Blotting, The graphs showed the relative density of LC3-II to LC3-I. (*n* = 4 for diabetic group, *n* = 5 for control group). **b** The protein levels of Beclin1 in Hippocampus, Cortex and Striatum were measured by Western Blotting, The graphs showed the relative density of Beclin1 to GAPDH. Note that the protein level of Beclin1 decreased significantly in the hippocampus of diabetic mice (*P < 0.05 *n* = 4 for diabetic group, *n* = 5 for control group). **c** The protein levels of p62 in Hippocampus, Cortex and Striatum were measured by Western Blotting, The graphs showed the relative density of p62/GAPDH

By using western blot analysis, we detected the expression levels of p62, LC3 and Beclin1 in hippocampus, cortex and striatum. Our results showed that the level of p62 had a increased trend in the STZ-induced diabetic mice hippocampus and striatum without markedly difference, and the ratio of the Beclin1 protein level decreased significantly in the diabetic hippocampus (Fig. 5). There was a upward trend of the ratio of LC3II/LC3I in hippocampus, cortex and striatum, but no statistically difference between the two groups.

## Discussion

Diabetes can produce long-term negative effects, such as high blood glucose, hypoglycemia, free radical-mediated oxidative stress, brain blood vessel reconstruction disorder and abnormal brain insulin [30, 31], thus, diabetes may represent a metabolic state in which neuroprotective and neuromodulatory effects of insulin in the Central Nervous System were disrupted [32], and causes brain damage. At present, the pathogenesis of diabetes related cognitive dysfunction is not clear. According to previous studies, diabetes related cognitive dysfunction consists of 2 types: One is vascular cognitive dysfunction which is caused by cerebrovascular disease; the other type shows apparent memory decline in association with atrophy in hippocampus and amygdala, but with no obvious cerebrovascular disease. In our study, we found that diabetic mice was prone to be accompanied by both cerebral small vessel disease and hippocampus atrophy, which was likely to be an important cause of diabetes related cognitive dysfunction, and maybe a predictor of cognitive impairment in patients with diabetes. This means that the pathogenesis of diabetic cognitive impairment include both of the two as showed before. Additionally, HFD/STZ-induced diabetic mice also showed apparent glucose uptake reduction in hippocampus and amygdala, indicating that vascular independent dementia might also be responsible for the cognitive decline.

In the present study, diabetes related cognitive impairment showed not only the decline of memory ability, but also apparent depressive symptoms. These emotional decline were corresponding to the glucose uptake reduction in hippocampus and amygdala, since the two brain regions were important for emotional integration. Current studies had confirmed that the hippocampal volume of depressed patients was significantly reduced [33–35], and the most serious damaged regions of depressed patients were hippocampus and amygdala [36]. Therefore, abnormalities in hippocampus and amygdala might induce emotional imbalance such as depression symptoms in diabetic mice. Few researches reported that diabetes induced reduction in metabolism of the amygdala, and our study suggested that the elderly diabetic mice had the dysfunction of glucose metabolism in the amygdala, which might be one of the pathological mechanisms of cognitive impairment. Therefore, these findings provided a new and interesting clue to find the intrinsic causes of memory impairment in patients with diabetes.

Autophagy played important roles of maintaining normal tissue and cellular homeostasis [37, 38]. Previous studies have showed that autophagy played important roles in the progression of metabolic syndrome, diabetes and other diseases. The pathogenesis of these diseases was, to some extent, related to the dysfunction of autophagic removement of damaged organelles [39]. It was found that the efficiency of autophagy function decreases with aging. Recent researches showed that type 2 diabetes could lead to autophagy dysfunction of vascular cells and neuronal cells, cause or aggravate cerebral vascular lesions and vascular dementia process, and eventually resulted dysfunction of memory [40–44].

The protein of p62, a protein associated with autophagosomes and degraded in the autolysosome, is commonly regarded as autophagy substrate protein [45, 46], as a mainly negative regulator of autophagy, activated mTORC1 inhibits autophagy induction through blocking of the initial inducer of autophagy [47, 48]. The p62 is the scaffold protein implicated in selective autophagy which is induced by the stress of cells. It plays an important role in the mechanism of diabetes. Beclin1 is not only a central positive regulator in the early stage of autophagy, but also one of the core proteins of autophagy, apoptosis and inflammatory reaction [49]. In recent years, scientists have discovered that Beclin1 protein levels decreased significantly in the brain regions of neurodegenerative diseases [49, 50], suggesting that Beclin1 may be involved in the cognitive dysfunction in Alzheimer’s disease. Our study found that the p62 protein expression in the brain tissue of elderly diabetic mice had a increased trend and the result meant that the autophagy in STZ-induced diabetic mice was downregulated. At the same time, the Beclin1 protein decreased markedly in hippocampus. Meanwhile, the result of LC3II/I showed that the diabetes group had a upward trend without statistical difference. These results indicating that the expression changes of p62 and Beclin1 might play different roles in the pathogenesis of autophagy. Based on our present study, we concluded that the autophagy was reduced in diabetic mice. Previous studies found that Akt plays a role in induced endothelial injury in diabetes [31], and that the NF-kB pathway is a mechanism of cerebral ischemia and hypoxia [51]. Next step, we will explore whether Akt and NF-kB pathway has an effect on diabetic cognitive dysfunction.

## Conclusions

The results of this study suggested that the elderly diabetic mice were prone to complicated with cognitive and affective disorders, which was probably caused by hippocampal atrophy, glucose metabolism disorders in the hippocampus and amygdala, and cerebral small vessel disease. Compared with normal aged mice, diabetic aged mice were apt to complicate cerebral small vessel disease with cognitive and affective disorders. Beclin1 mediated autophagy might play an important role in the process of its occurrence and development. The intrinsic molecular mechanism still need further deep-going research in the future.
